# On the Preservation of the Deciduous Teeth

**Published:** 1876-02

**Authors:** N. Marshall Burkholder

**Affiliations:** Harrisonburg, Va.


### ARTICLE II.

### *On the Preservation of the Deciduous Teeth.*

BY N. MARSHALL BURKHOLDER, D. D. S., HARRISONBURG, VA.

Read before the Virginia Dental Association.

The first ten years of the child is too apt to be a period of neglect, as it respects the teeth. The fact that the first teeth are soon lost, or rather must soon give place to others accounts, I suppose, in some measure for the little attention they have commonly received from dental practitioners and from parents as well.

It is very easy, however, to get into the habit of crediting the general public with very much more knowledge on these subjects than they actually possess; and to forget that a far more important reason of the neglect of the teeth of children is found in a want of acquaintance with those facts of physiology which proclaim the importance of their preservation.

Very many parents, in my experience, possess only enough acquaintance with this general subject to know that their children "*will get other teeth*" when the milk teeth are gone. They have no idea of a regular period for their appearance, which will not be hastened, but rather retarded, by the untimely loss of the first teeth. They have never given any thought to a consideration of the conditions necessary to the formation of the permanent teeth, or to perfect nutrition and vigorous growth. They have scarcely noticed an increased number in the second set. How common the dialogue in every dental office with regard to the first permanent molar: Parent referring to child—"He will get another tooth there." Dentist—"No; that is one of the second set." Parent—"Why he has never lost that tooth yet."

The great masses of the people know nothing of these things. It is not because they are lacking in intelligence that they suffer so much from carelessness and neglect, but, because they are so busy in the pursuits of life that they cannot spare time, they imagine to read and study the laws of hygiene and physiology. They have all the indifference of our grandfathers, without however, having our grandfathers teeth. And what is more, they are not to be expected to inform themselves on these subjects until their importance is pointed out to them, and their attention is in some degree awakened.

There has undoubtedly been great neglect on the part of the dental profession, as a whole, in this very important sphere of our labors. The attention of the people in many communities has scarcely ever been called to the importance of the preservation of the deciduous teeth; to the importance of a careful observation of the mouth during the entire period of the eruption of the permanent teeth, and of the timely correction of incipient irregularity.

It is to this matter I wish particularly to direct the attention of our Association. I regret that I cannot reach the ear of every practitioner in Virginia. For it often happens that the members of our Association, least of all, need these exhortations. They are commonly alive to their duties, and very often far in advance of the speaker who is urging them forward. But let me say, if the dental profession would discharge its obligations in the highest sense, it must instruct as well as practice. We must be teachers as well as surgeons. I fear exceedingly that, as a people, we are neglecting the teeth of our children, and I believe that dental surgery will have to look and labor more in this direction in the future, than it has ever done in the past.

It is gratifying, however, to note the fact that so many at this day are turning their attention to this class of teeth. The inquiry is arising in many minds, "Is there not something more to be done here, and how shall we reach the children." It is beginning to be perceived that all truth is correlated; that the beginning of things are usually the most important. No subject, however trivial or slight its relation to other subjects may at first blush appear to be, is to be despised by him who would attain those truths which in anywise concern the happiness of man.

The period of childhood is so much characterized by bloom, and promise, and rapid growth, that it is hard to think of a necessity of guarding against decay. Besides it has never been customary to give any attention to their teeth. "Whoever heard of such a thing," occurs to every one. But the ruling fact of a greatly inferior structure of the teeth of children of to-day, as compared with their parents or grandparents, stares us in the face, and necessitates that care and attention which our ancestors could do without.

Not very long ago it was exceedingly rare to find a dentist who was much exercised about the teeth of children. But may we not reasonably hope we are fast nearing the time when the dental profession, as a whole, shall enter this wide field; and when the children now the subjects of universal mighty effort in the domains of the heart and head, will have enlisted in their behalf those efforts which are calculated to promote their physical well being and therefore their comfort and happiness.

I lay down the proposition, which will commend itself to every one, that if nature intended the deciduous teeth to be a substitute for the teeth which should be permanent, until the system is able to bring forth these permanent teeth, then it is our duty to preserve them as long as they are wanted, according to this design of nature. And I venture to assert on the principle that no design of nature, or function of an organ can be thwarted or destroyed without influences more or less hurtful to other organs, and to the general system; that the invasion by diseases of these organs (the first teeth) and the premature loss of their function is to the no slight detriment of the system. The principal function of the teeth is mastication. The preparation of the food for digestion—for absorption and assimilation lies at the very beginning of the process of healthy growth, or rather of the conditions necessary thereto. We perceive the necessity of requiring that the infant taken from its mother's breast, as it begins an independent existence, should be fed on food which requires no teeth for its preparation for the stomach. Food which has been elaborated through the operation of the teeth of another has hitherto been its support. As the teeth are erupted its ability to partake of more solid food is correspondingly increased. Before its second year, however, the child has no business at the table with the rest of the family. About the end of the third year it may, if taught by considerate parents to masticate well, subsist upon any solid food, the deciduous, or temporary, the first or milk teeth as they are variously termed, being now usually all in place.

We are now entering upon a critical period in the history of the child in respect of its physical development. This is peculiarly the formative period. The most rapid growth of brain occurs between the ages of three and ten. I believe it to be true in a physical as in a moral sense, "Give me the child at three years old, and when he has lost his milk teeth you may make what you please of him." It is the period of greatest impressibility. The slightest force gives new direction to the currents of life and character; the slightest impediment may hinder and clog its delicate mechanism. Inferior mastication here makes bad digestion, and this impairs nutrition, and nervous disorder ensues with lowered vitality. Such a state childhood's innumerable ailments find most readily; and here disease reveals with least opposition. If, however, all the offices of the various organs continue to be healthily performed, if there be exercised a judicious care to favor a uniform and harmonious development of the physical and of the nervous systems, counteracting here and promoting there such prominent inherited tendencies as are observed, we may expect a well developed, well balanced organization, health vigor and happiness.

I frankly admit that I have here introduced a saving of great potency. I believe that that acute observer, Prof.

Kingsley, (witness his September article "*American Journal of Dental Science*,") has pointed out to us some important facts in this connection, and further that he has discovered to us the method of approach to others. It has for many years been the declared opinion of the first educators, and of the first medical men, that the intellect of the child should not be pushed; that the physical system should have fair play between the fifth and tenth years. In other words, that a solid substructure of body should be first provided, and on this erected the brain.

We have but to look about us with the most casual glance to perceive that the tendency to excessive nervous development is on the increase. Why, sir, there is such a thing as attaching too much importance to education; and this is one of the evils of it. In the sphere of religious effort intellectual endowments sometimes come to be considered before spiritual endowments. "Mind over matter" has been so persistently hung up in letters of green on brilliant commencement occasions, that many a young man has half wished himself out of the body, and has thought it matter for praise that he has actually reduced his body to the lowest minimum.

This atmosphere has been so much breathed that it is to be feared "mind" has sometimes come to appear to be "over matter," like the figure of liberty on our Virginia coat of arms is over the tyrant who lies crushed and despised at her feet. Everything has its limits; it will not do to disturb the natural relation of things. Mind is over matter, but still connected with it. And our bodies are not to be lightly esteemed They evince the masterly workmanship of a Master's hand. They bear the stamp of divinity. And it becomes our duty, our first duty as between the physical and the intellectual, to preserve this body and each and every of its members. Especially is it seen our first duty when we consider that through the medium of the body the mind manifests itself.

Except under infatuation the human mind always acts upon this conclusion. "Self preservation is the first law of nature." But the spirit of infatuation is abroad. Throughout the entire frame-work of modern society, in all its channels, and intricate underrents, everything is on a *strain.*

I am reminded in this connection of the humorous and original Prof. Thos. E. Bond, who, in speaking about what he termed the "emasculate practice of the times," in displacing the lancet, and in its reliance upon that great class of remedies known as stimulants, remarked that the present practice seemed to be based upon the theory that "*Life is a forced state, and whiskey a forcer.*"

An undue development of the brain and nervous system occupies an important place in relation to irregularity of the teeth, imperfect tooth structure, and to the deterioration of those qualities of the human frame which capacitate man for endurance. That relation is entitled to be declared one of the recognized facts of dental science of to-day. It affects all children in precise correspondence, (other things equal,) with their trangression of the laws of normal development, in acceding to the wrong demands of civilization, the natural tendency of which is in laboring for the development of the mind, not to give enough thought to the highest development of the body.

Premature mental exertion dwarfs the physical system by robbing it of blood and of nervous force, thus impairing the digestive and nutritive powers which have to do with growth. But *mastication* being a condition upon which digestion depends, if it be wanting or imperfect, it will, by so much, inevitably tell upon the digestive capacity, and in the general results of nutrition and of organic structure, the brain itself included. In this way the digestive apparatus is overworked ; in the former it is robbed of its power. In both, its powers are diminished. In the one case you overstrain the brain, which draws upon the entire system for its support; in the other you overstrain the stomach, producing general relaxation or diminution of vigorous tone.

These are sober facts which should be read and pondered by parents everywhere. It is no less our duty to bequeath to our posterity a vigorous body than a vigorous mind. In truth the highest effective capacity of mind, other things equal, must ever be linked to the highest physical development.

Now if we consider the earnest demand for vital power in the growing child, coming from very rapidly forming tissue, and organ, we begin to perceive the serious nature of the least unnatural action or derangement of the function of digestion and other conditions of supply. And then with this, taking into view the tendency to excessive nervous development, the results of which,—an abatement of the vigor of the nutritive forces, and a lowering of the forming constitution—are increased and intensified in force by inheritance from generation to generation, it seems to me it ought to be considered all-important to preserve in perfect order the masticatory organs; to preserve always all the conditions directly concerned in the up-building of the physical system, but especially when an influence so potent is operating to hinder and weaken and drain away its powers.

The deciduous teeth form a most necessary part of nature's laboratory in a most critical period of her labors. They should receive the attention of parent and dentist, until one after another, they are displaced by their successors. The very gradual method of natural displacement which makes the period of their disappearance scarcely felt as it regards the power of mastication should teach us that nature foresaw she could not spare them. It ought to be regarded quite as serious a matter to lose a deciduous tooth as a permanent tooth. It ought to be held an important matter, not to be neglected, to have a child continuously refusing to chew on one side of the mouth on account of the presence of a diseased and sensitive tooth. Who has not seen it? Permanent injury to all on that side ensues. Apart from the suffering and its general depressing effects, affected glands, vitiated saliva, imperfect digestion, more or less, make up a sad and what is worse, a useless picture. Or if the teeth are lost, by so much precisely as the power of mastication is reduced and its proper performance hindered, will the process of nutrition and healthy, vigorous, perfect structural formation be impaired as the ultimate result.

Who is prepared to demonstrate it a small matter to have an inferior digestion during this period of peculiarly rapid development, unattended though it be by any disorders visible save those affections of the blood and skin so common to the young.

During this period of rapid growth of bone, and muscle and nerve, the formation of the permanent teeth is also steadily progressing. The forming teeth seem to possess a peculiar sensitiveness in the presence of constitutional disorder. Take, for instance, the care of atrophy of the teeth. Where in other structures of the body are the corresponding marks of those constitutional disturbances which produce it. The teeth seem to be developed on the remotest limits of vital flow, and the delicate threads and silken fibres of their existence are made to tremble and pale at the slightest ebb.

If good mastication and good digestion be important at anytime,--if the conditions necessary to the most perfect nutrition and most healthy growth are important at any time of life, are they not especially so during this period? "The child is father to the man." What if nature, kind mother, has furnished the child these teeth with which it may, as it were, work out for itself others whose structure shall be so much more enduring as the conditions under which they are formed are superior to the conditions under which the first were formed. How lamentable the reflection that in so many instances these conditions are permitted to be so little superior to the first. The buoyant forces of nature, as they go on to fill up the stature and complete the structures of the body are not easily overcome; but the secret and destructive influences they experience are not the less real because they are unseen.

The presence of the deciduous teeth and the proper performance of mastication has its natural relation to the healthy formation of the permanent teeth, and we are bound to treat the deciduous teeth with this fact in view.

Considerations like these should require nothing supplemental to discover to us our duty. It should be added, however, that the preservation of these teeth has an important bearing upon the natural and orderly placing of the permanent teeth. Their premature loss in certain contingencies, occurring in almost every mouth, is followed by *irregularity* and *crowding* of the permanent teeth. Every parent should seek to avert this calamity. For a crowded condition of the teeth is one of the great predisposing causes of decay.

Not the least of the benefits to be derived from the preservation of the deciduous teeth is the habit of cleansing the teeth with brush and quill daily. Perhaps the most lamentable want of our times, in connection with this whole subject, is *the habit of cleansing the teeth.* Habit, to be firmly fixed, must be begun early. *It has come to this, that every wise mother must make it a part of her duty to see to it that her children are taught to use the Brush as soon as they are taught to wash their faces.*

The habit of giving attention to the teeth thus early begun, coupled with the necessary instruction and advice, serves not only to fix impressions of their frailty, but also of their value. And thus a strong sense of duty, the safe guard against neglect, is early attained and never lost. The brush should be used at least morning and evening,—deferred in the evening until just before going to bed. The teeth are thereby freed from whatever may have been taken into the mouth after supper, fruits, etc., and acid secretions. No money pays better than that put into tooth brushes. But the brush must be aided by the quill. There are points in perhaps every mouth, where solutions of food, or other acidulated matters cannot be disturbed with the brush. The quill breaks up these nests on proximal surfaces. The use of brush and quill is not a burden; it becomes a matter of comfort, and one does not feel well without them.

I have a little boy, aged 9, who has been using the brush daily since he was four years old. Two of his first teeth have been filled, and none of them have been lost except in the natural process of displacement.

There is too much indifference to the wants of children because they are children. Children are almost universally allowed to neglect their teeth because of a lack of decision in parents, in enforcing the regular use of the brush. And in many cases the dread of a small expense,—small in the majority of cases where the habit of cleansing is observed, is the obstacle in the way of their preservation.

By urging the habit of cleansing, by frequent examinations, by judicious separations, and by fillings, it may be with the cheaper materials, with very short sittings, we must preserve the children's teeth. It requires patience, gentleness, and strict truthfulness to treat with children. And what is more, the decided support of the parent.

As the people are made acquainted with these particulars they become more careful of their teeth, and of the teeth of their children. Our profession should endeavor to gather up the truth on these subjects, and disseminate it. This, if I understand it, is one of the benefits resulting from Associations like this. There is great sale for small, judiciously prepared, popular treatises on these subjects. Education is the one idea of the day. While the popular mind is being drawn out in other departments of useful knowledge we must provide the needed information in this important field.

For forty years our profession has labored with unabating effort to convince the world of the importance of the teeth, and of the necessity of educated and skilled men to assist the people in preserving them. You have successfully demonstrated it to men of science and to the masses alike. You have humbly, quietly, and yet resolutely, and by the effective aid of many of the medical profession, demonstrated the importance and the possibility of preserving the teeth. You have lifted it from a position of vague uncertainty and non-attention in which it was regarded, and have shown that the teeth stand closely and influentially related to the human system.

But we have work yet before us. In the heat of the contest there seemed to be little time to attend to the children. We thought little about them, and did less for them. The great public see now the importance of preserving *their* teeth. *But they do not yet generally understand that we have a mission to their children.* The time has come when we must advocate the cause of the children.

It is the high duty of the dental and medical professions alike to endeavor to acquaint men with the great fundamental laws of health and of disease, and with the facts and conditions upon which disease in general depends.

The diffusion of much knowledge tends not only to diminish the accidents of disease, but, through an intelligent apprehension of disease once begun, secures more apparently the needed professional assistance, provides against relapses and thus multiplies the success, and enhances the character and dignity of the healing art.

